# A Comparative Study of Biological Characteristics and Transcriptome Profiles of Mesenchymal Stem Cells from Different Canine Tissues

**DOI:** 10.3390/ijms20061485

**Published:** 2019-03-25

**Authors:** Xiao-Shu Zhan, Saeed El-Ashram, Dong-Zhang Luo, Hui-Na Luo, Bing-Yun Wang, Sheng-Feng Chen, Yin-Shan Bai, Zhi-Sheng Chen, Can-Ying Liu, Hui-Qin Ji

**Affiliations:** 1 School of Life Science and Engineering, Foshan University, Foshan 528231, China; m18306618269@163.com (X.-S.Z.); saeed_elashram@yahoo.com (S.E.-A.); ldz520fs@163.com (D.-Z.L.); 13794646453@163.com (H.-N.L.); xuefei200403@163.com (Y.-S.B.); czsfight@163.com (Z.-S.C.); liucy3032@163.com (C.-Y.L.); 13928293206@163.com (H.-Q.J.); 2 Faculty of Science, Kafrelsheikh University, Kafr el-Sheikh 33516, Egypt

**Keywords:** canine, mesenchymal stem cells, biological characteristics, RNA-sequencing

## Abstract

Mesenchymal stem cells (MSCs) are the most promising seed cells for cell therapy. Comparing the biological and transcriptome gene characteristics of MSCs from different sources provides an important basis for the screening of clinically used cells. The main purpose of this experiment was to establish methods for the isolation and culture of MSCs from five different canine sources, including adipose tissue, bone marrow, umbilical cord, amniotic membrane, and placenta, and compare biological and transcriptome characteristics of MSCs, in order to provide a basis for the clinical application of canine MSCs. MSCs were isolated from Chinese pastoral dogs, and the following experiments were performed: (1) the third, sixth, and ninth generations of cells were counted, respectively, and a growth curve was plotted to calculate the MSC population doubling time; (2) the expression of CD34 and CD44 surface markers was studied by immunofluorescence; (3) the third generation of cells were used for osteogenetic and adipogenic differentiation experiments; and (4) MSC transcriptome profiles were performed using RNA sequencing. All of the five types of MSCs showed fibroblast-like adherent growth. The cell surface expressed CD44 instead of CD34; the third-generation MSCs had the highest proliferative activity. The average population doubling time of adipose mesenchymal stem cells (AD-MSCs), placenta mesenchymal stem cells (P-MSCs), bone marrow mesenchymal stem cells (BM-MSCs), umbilical cord mesenchymal stem cells (UC-MSCs), and amniotic mesenchymal stem cells (AM-MSCs) were 15.8 h, 21.2 h, 26.2 h, 35 h, and 41.9 h, respectively. All five types of MSCs could be induced to differentiate into adipocytes and osteoblasts in vitro, with lipid droplets appearing after 8 days and bone formation occurring 5 days after AD-MSC induction. However, the multilineage differentiation for the remaining of MSCs was longer compared to that of the AD-MSCs. The MSC transcriptome profiles showed that AD-MSC and BM-MSCs had the highest homology, while P-MSCs were significantly different compared to the other four types of MSCs. All the isolated MSCs had the main biological characteristics of MSCs. AD-MSCs had the shortest time for proliferation, adipogenesis, and osteogenic differentiation.

## 1. Introduction

Mesenchymal stem cells (MSCs) are a type of adult stem cells derived from the mesoderm. MSCs were first discovered by Friedenstein and his colleagues in mouse bone marrow cultures half a century ago [1]. Later, the cells were isolated from a number of adult tissues, including adipose tissue [2], pulp [3], and postpartum discarded tissue, such as umbilical cord [4,5,6], placenta [7], amniotic membrane [8], and cord blood [9]. MSCs lack unique cell membrane surface markers, and can be identified by three biological characteristics: adherent properties, a series of cell-specific expression of cell surface markers, and multilineage differentiation potential [10]. The rise in high-throughput sequencing technologies in the past few years has made it possible to understand MSCs at the molecular level, and further tap the potentials of MSCs.

Humans have owned dogs as companions for thousands of years. With improving living standards worldwide, dog health has improved, and the life span of dogs has increased. Improved dog health and longevity can be ascribed to advanced diagnostic and veterinary treatment procedures [11,12,13]. In addition, dogs have become an ideal animal experimental model, because they have developed nervous and digestive systems [14,15,16], as well as a number of physiological characteristics that are similar to humans [17]. As a result, dogs are often used in drug development clinical trials [18,19]. Current traditional medical treatments have a poor effect in curing numerous dog diseases and injuries, including nerve damage, kidney damage, hepatitis, and diabetes. Therefore, the scientists are constantly exploring new medical tools [14,20,21,22,23]. The emergence of stem cell therapy brings new hope for the treatment of these diseases [24,25]. The homing and low immunogenicity of MSC make them ideal seed cells for stem cell therapy [26]. However, MSC application-based research has been mostly from human and murine sources, and there has been less research on dog MSCs. What remains unclear is whether dog MSCs have the same biological characteristics as other sources. In addition, previous studies have addressed two or three different sources of MSCs; however, this study was conducted to systematically compare the most common five different sources of MSCs. Therefore, accelerating the study of the biological characteristics of canine MSCs will provide a theoretical basis for the clinical selection and use of MSCs, and bring new hope for the veterinary field and translational medicine. MSCs not only have repair and therapeutic effects in viral and injurious diseases, but also have diagnostic and therapeutic effects in metabolic diseases, such as obesity and pregnancy-related diseases [27,28,29]. The overall aim of this study was to investigate growth characteristics and the proliferative capacity of MSCs derived from adipose, bone marrow, umbilical cord, placenta, and amnion. Furthermore, adipogenic and osteogenic differentiation, as well as the transcriptome profile of MSCs was studied to provide baseline data on canine MSCs, which may be used in follow-up clinical cell therapies.

## 2. Results

### 2.1. Isolation and Primary Culture of Mesenchymal Stem Cells

**Figure 1:**
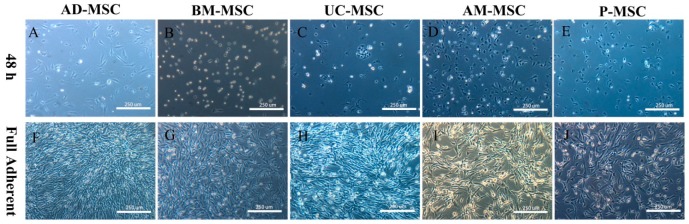
Adherence and primary growth of five types of mesenchymal stem cells (MSCs) from different sources (100×): adipose mesenchymal stem cells (AD-MSCs), placenta mesenchymal stem cells (P-MSCs), bone marrow mesenchymal stem cells (BM-MSCs), umbilical cord mesenchymal stem cells (UC-MSCs), and amniotic mesenchymal stem cells (AM-MSCs). Morphology of mesenchymal stem cells in primary culture for 48 h (**A**–**E**). Status of mesenchymal stem cells in primary culture to obtain full adherent (**F**–**J**).

After 48 h of primary culture, a small number of adhered cells could be observed in all of the five types of cells. Initially, the cell morphologies were uneven, showing short spindles or polygons (Figure 1A–E). The cell-forming clones were scattered in the bottom of the dish. Adipose mesenchymal stem cells (AD-MSCs) reached 90% confluence on the fifth day, while the other cells reached confluence around the tenth day (Figure 1F–J).

The cell morphologies were subsequently fibrous or fusiform. After the passaging of the cells, the proliferation rate of the cells increased exponentially, and the cell morphologies were relatively uniform (i.e., they could be considered pure MSCs). Therefore, P3 cells were used in the subsequent experiments.

### 2.2. Growth Curve and Population Doubling Time of Mesenchymal Stem Cells

**Figure 2:**
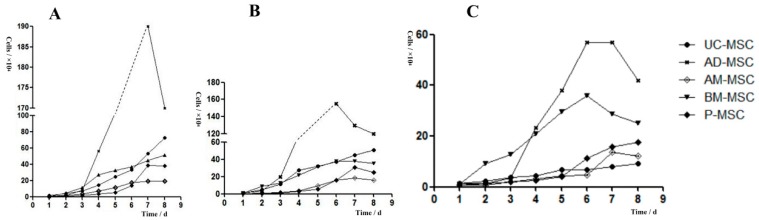
The growth curves of MSCs: (**A**) third generation, (**B**) sixth generation, and (**C**) ninth generation.

As can be seen from Figure 2, the proliferation of MSCs was not significant at 1 to 2 days after passage. However, MSCs entered the logarithmic growth phase from the third to the sixth day and entered the plateau phase at the seventh day. According to the growth curve of P3 cells, the mean population doubling times of AD-MSCs, placenta mesenchymal stem cells (P-MSCs), bone marrow mesenchymal stem cells (BM-MSCs), umbilical cord mesenchymal stem cells (UC-MSCs), and amniotic mesenchymal stem cells (AM-MSCs) were 15.8 ± 0.318 h, 21.2 ± 0.629 h, 26.2 ± 0.242 h, 35 ± 0.588 h, and 41.9 ± 0.954 h, respectively. There were significant differences among the groups (*p* < 0.01). The population doubling time (PDT) of AD-MSC was significantly lower than that of the other four types of MSCs, indicating that AD-MSC has the fastest cell growth rate and the strongest cell proliferative ability when cultivated in vitro.

### 2.3. Mesenchymal Stem Cell Immunofluorescence Results

**Figure 3:**
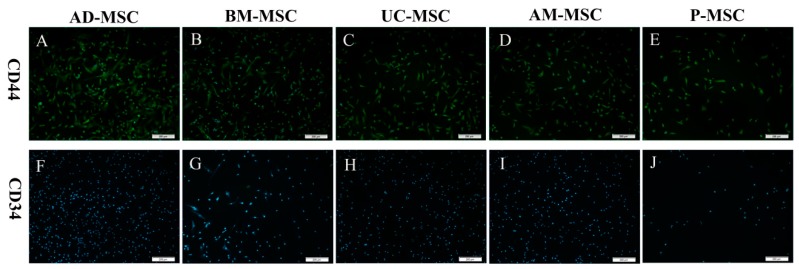
Immunofluorescent identification of five MSC types (100×). All five types of MSCs positively express CD44 surface markers (**A**–**E**) and negatively express CD34 surface markers (**F**–**J**).

The immunofluorescence identification revealed that all five MSC types expressed CD44 (Figure 3A–E), but not CD34 (Figure 3F–J).

### 2.4. Adipogenic and Osteogenic Differentiation Results

**Figure 4:**
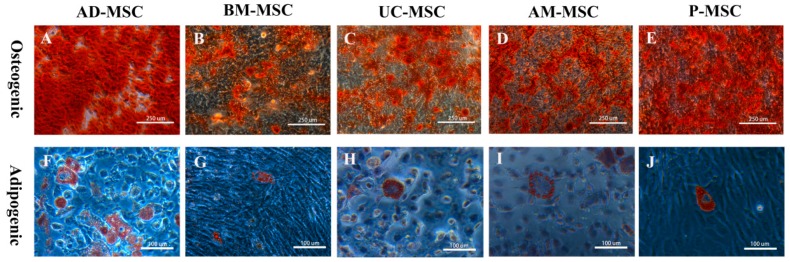
Results of osteogenic and adipogenic differentiation of the five types of MSCs. (**A**–**E**) Alizarin Red staining, 100×; (**F**–**J**): Oil Red O staining, 200×.

After three days of osteogenic induction, the shapes of the cells changed from fibers to polygons and scales. Calcium nodules appeared in the AD-MSCs, P-MSCs, UC-MSCs, AM-MSCs, and B-MSCs after 5, 7, 8, and 9 days of induction, respectively. All five types of MSCs were stained with alizarin red on the tenth day of induction. All MSCs showed red mineralized deposits (Figure 4A–E).

After adipogenic differentiation, the changes in the five types of MSCs were similar, and the cells had gradually shortened and rounded from their original typical fibrous shape. Initially, there were more dead cells in the induced differentiation. However, after a later stage of cell differentiation they became stable.

Lipid droplets were observed in AD-MSCs and BM-MSCs after 8 and 12 days of induced differentiation, respectively After two weeks of induced differentiation, small scattered droplets were observed in UC-MSCs, AM-MSCs, and B-MSCs. After one more week of induction, the lipid droplets became larger and their number increased. Oil red O staining showed that the lipid droplets were stained red (Figure 4F–J). The above results indicate that AD-MSCs have the fastest growth rate of the tested cells in osteogenic and adipogenic differentiation.

### 2.5. Expression of Surface Marker Genes on Five Types of Mesenchymal Stem Cells in Dogs

In this research, the fragments per kilobase of exon model per million reads mapped (FPKM) values of the five types of MSCs (under the same experimental conditions) were considered as the final reference values. The results, as shown in Table 1, indicate that canine MSCs not only express three surface markers—CD73, CD90, and CD105—as defined by the International Society for Cell Therapy (ISCT), but also express CD13, CD44, CD49a, CD54, CD140a, CD140b, and MHC I (FPKM > 1), but not CD11a, CD11b, CD14, CD19, CD33,CD34, CD45, CD86, CD 146, CD 271, or MHC (FPKM < 1). However, due to the lack of suitable antibodies, some of these genes have not been detected at the protein level.

### 2.6. Cluster Analysis of Differentially Expressed Genes

**Figure 5:**
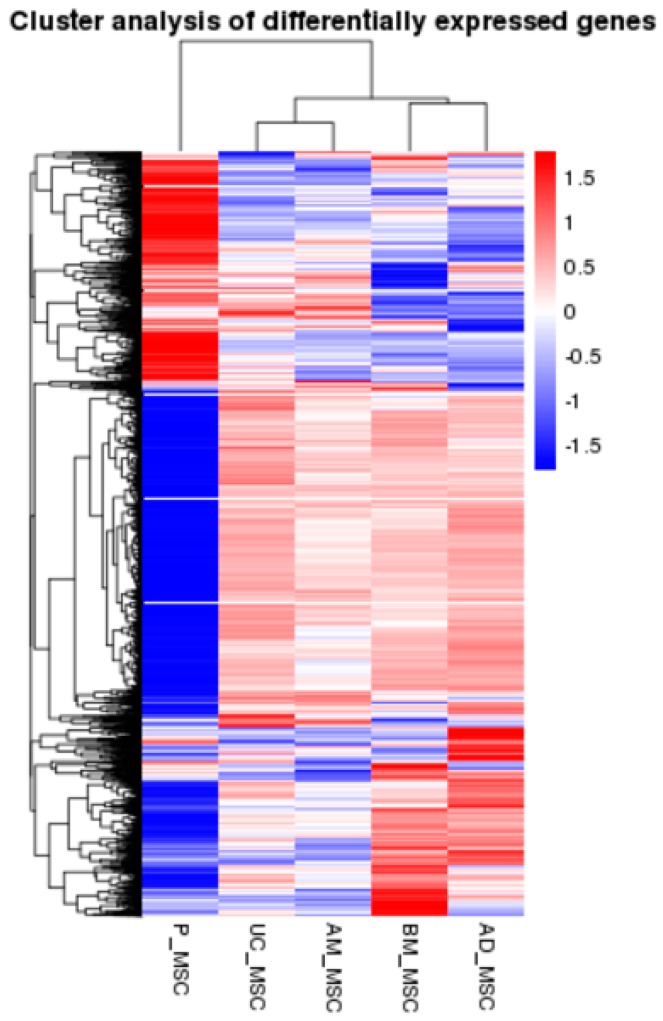
Dendrogram and unsupervised hierarchical clustering heat map (using Euclidean distance) of gene expression, based on the log ratio fragments per kilobase of exon model per million reads mapped (FPKM) data. Each column represents an experimental condition; each row represents a gene. For differentially expression genes, their log2 (RPKM) will be clustered. Red indicates up-regulation, and blue indicates down-regulation. The vertical distances on each branch of the dendrogram represent the degree of similarity between gene expression profiles of the five MSC groups.

This study investigated the changes in gene expression of the gene expression profile of five types of MSCs. The unsupervised hierarchical clustering of our complete RNA-Seq transcriptome data was performed to compare the expression pattern of differential expression genes in the five groups. The clustering heat map reveals that UC-MSC and AM-MSC, as well as BM-MSC and AD-MSC gene expression levels were similar, while P-MSC gene expression levels were significantly different from those the other four types (UC-MSC, AM-MSC, BM-MSC, and AD-MSC) (Figure 5).

### 2.7. Transcriptome Sequencing Analysis

The transcriptome sequencing results (see Supplementary Material) showed that perinatal-derived MSCs (AM-MSCs, UC-MSCs, and P-MSCs) and body-derived MSCs (BM-MSCs and AD-MSCs) shared 94 up-regulated and 150 down-regulated genes, respectively. Typical differentially expressed genes are listed in Table 2.

### 2.8. Validation of Transcriptome Results by Quantitative Reverse Transcriptase-PCR

**Figure 6:**
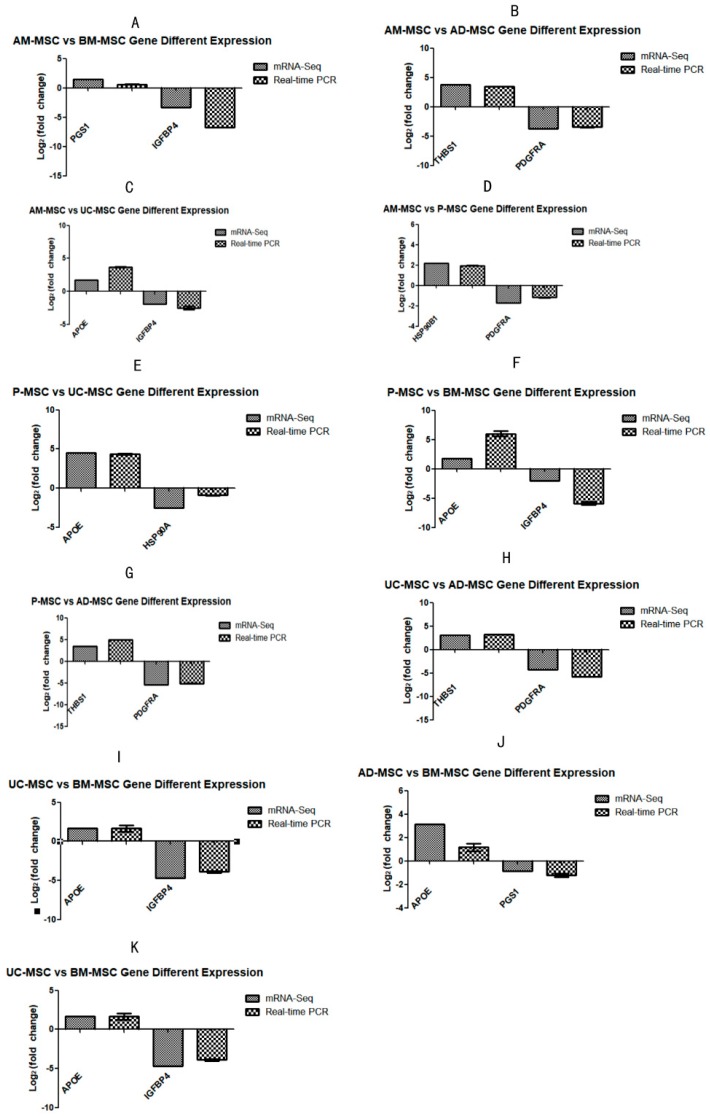
Validation of RNA-Seq data by employing qRT-PCR for the expression of Apolipoprotein E (APOE); heat shock protein 90, alpha family class A member 1 (HSP90AA1); insulin-like growth factor binding protein 4 (IGFBP4); thrombospondin 1(THBS1); and platelet-derived growth factor receptor alpha (PDGFRA). *X*-axis: gene name; *Y*-axis: log2 fold change in gene expression in each group (**A**–**K**).

Differential and non-differentiated genes in each group were selected for qRT-PCR analysis to validate transcriptome data, and the results showed a consistent trend with Illumina sequencing data, indicating the reliability of comparative analysis of our transcriptome (Figure 6).

## 3. Discussion

Basic research on stem cells has made the development of novel biopharmaceuticals, cell therapy, regenerative medicine, and tissue engineering possible [5]. Although pluripotent embryonic stem cells isolated from early embryos have the greatest potential, they face enormous technical challenges and ethical dilemmas [30,31]. To circumvent technical and ethical challenges that are associated with embryonic stem cells, adult MSCs have become an attractive alternative in stem cell research, because of their ability for self-renewal and multi-differentiation. Compared with embryonic stem cells, MSCs have multiple sources and lower immunogenicity, and avoid ethical and moral controversy.

In recent years, it has been found that adipose-derived MSCs have outstanding growth performance and differentiation potential [32], while MSCs derived from postpartum discarded tissues, such as the umbilical cord [4], placenta, and amniotic membrane are readily accessible with low cellular immunogenicity, and can be acquired from multiple sources. Therefore, MSCs have become a hot area of research [5,33]. Although a number of published reports on the biological characteristics of adipose-derived, umbilical cord, placenta, and amniotic membrane MSCs are available, the publications are mostly about human-derived MSCs [7,34]. The fact is that these cells were derived from different individuals and cultured in the different growth conditions, which have different influences on the characteristics of MSCs. To avoid problems associated with MSCs from different individuals that are cultivated in different conditions, in this study we successfully isolated MSCs from five types of canine tissues, including fat (adipose tissue), bone marrow, umbilical cord, amniotic membrane, and placenta (AD-MSC, BM-MSC, UC-MSC, AM-MSC, and P-MSC, respectively) from the same dog. The biological characteristics and gene expression of the five types of MSCs were compared.

All the five types of MSCs in this study were fibrous adherent cells. Since there is controversy regarding the identification of MSCs in the international community, this study used a series of criteria developed by the International Society for Cell Therapy (ISCT) to identify the isolated cells [35]. According to the immunofluorescence staining results, all the five types of MSCs expressed the hyaluronate receptor CD44, but did not express the hematopoietic stem cell marker CD34; in addition, all of MSCs were induced into adipocytes and osteoblasts. Therefore, cells isolated from these five types of tissues were identified as MSCs. A transcriptome profile has found that canine MSCs not only express CD73, CD90, and CD105, which is consistent with the internationally recognized characteristics of MSC surface protein expression [36], but they also express CD13, CD44, CD49a, CD73, CD140a, and CD140b, which is in accordance with the surface markers of human-derived mesenchymal stem cells [10,37,38]. In addition, our study found that canine MSCs express CD54 and MHCI surface markers, but do not express CD8, CD33, CD29 and MHCII surface markers, which is consistent with previous studies on canine MSC results [39,40,41]. Interestingly, our study showed that canine MSCs did not express CD146 and CD271, in contrast to a report by Boxall et al. [42], which showed that CD146 and CD271 could be produced by human bone marrow mesenchymal stem cells. BM-MSCs were used as the gold standard for the comparison of the biological characteristics of other MSCs [32]. These controversial results may be ascribed to differences in the expression of MSC surface markers in different species. This study has shown that to a certain extent, there are differences in the growth performance and differentiation potential of MSCs from different tissue sources in vitro. According to the growth curve analysis, as the number of passages increased, both the growth rate and the number of MSC cells decreased, which is in line with those of previous reports [32,43].

We compared MSCs from the same generation, but from five different sources, and found that AD-MSC cells had the highest proliferation activity in vitro. On average, the number doubled every day. Additionally, MSCs still sustained good activity after 15 passages. Our results are similar to those obtained by Keith A et al. [32,44]. Our results showed that AD-MSC cells were the smallest volume among the five types of MSCs, implying that relatively more cells are needed by AD-MSCs to achieve saturation density in the proliferation process. Furthermore, the growth curve showed that AD-MSC cell density exceeded other MSCs, and was mainly associated with the high expression of AD-MSC in cell division-associated protein 8 (CDCA8) and cyclin B2 (CCNB2) [9]. Additionally, the expression of CDCA8 and CCNB2 in AD-MSC was higher than that of MSCs from other sources, but whether this may be the reason for the rapid proliferation of AD-MSC remains to be further explored. In addition to the ability of proliferation, differentiation is another important feature of MSCs. Differentiation is characterized by dramatic changes in the size, morphology, membrane potential, and the metabolic activity of cells [45].The results of this experiment showed that the five types of MSCs can be differentiated into adipocytes, with AD-MSC having a significant advantage for lipid droplets to form and increase in numbers. The results of osteogenic differentiation showed that AD-MSC, followed by P-MSC, needed the shortest amount of time to form calcium nodules. Moreover, all the five types of MSCs had a high rate calcium nodule formation.

Based on the results of transcriptome sequencing, the five types of MSCs were divided into two groups: body-derived MSCs (bone marrow and fat MSCs) and perinatal-derived MSCs (placenta, amnion, and umbilical cord MSCs). Analysis and comparison of differential gene expression between the two groups of MSCs indicated that different genes may be involved in different biological processes and signaling pathways. Differential gene analysis showed that 94 genes and 150 genes in body-derived MSCs were up-regulated and down-regulated, respectively, in comparison between the two groups. Typical genes with down-regulated expression were *APOE*, *CDKN1A*, and *THBS1*, and those with up-regulated expression included *TFPI2*, *DPT*, and *FBN1*. Liu et al. have demonstrated that apolipoprotein APOE can process and transport fatty acids to nerve cells to protect neurons [46]. Overexpression of the cyclin-dependent kinase inhibitor 1A (CDKN1A) causes cell cycle arrest, thus inhibiting cell proliferation [47].It has been proven that THBS1 plays a role in platelet aggregation and angiogenesis, while PHLDA1 has been shown to be involved in the anti-apoptotic process of IGF1 [48]. DIO3 regulates the circulating fetal thyroid hormone concentration during pregnancy, and thus prevents the premature exposure of fetal tissue to high levels of thyroid hormone [49]. Due to the special function of the perinatal tissue to breed the fetus, DIO3 is highly expressed in the perinatal-source MSCs. The product of the H19 gene is a long non-coding RNA that acts as a tumor suppressor. The TGFBI protein is induced by transforming growth factor-β, and can inhibit cell adhesion [50]. Mutation of this gene is associated with various types of corneal dystrophy. We observed that perinatal-derived MSCs, compared with body-derived MSCs, had lower proliferation activity and lower viscosity, which means they are more suitable for the treatment of nerve injury, thyroid hormone abnormalities, and so on. They also promote the speed of coagulation and angiogenesis, and are anti-apoptotic.

Among the up-regulated gene expression in body-derived MSCs, the *TFPI2* gene has been identified as a tumor suppressor gene in several types of cancers [51]; DPT is a kind of extracellular matrix protein, which is considered to be the communication link between the surface and extracellular matrix environment of dermal fibroblasts. The control of TGF-beta bioavailability and the calibration of TGF-beta and BMP levels can adjust osteoblast maturation [52], which suggests that the high osteogenic differentiation efficiency of body-derived MSCs in previous studies may be connected with their own high expression. S100A16 is a kind of calcium-binding protein that binds one calcium ion per monomer [53]. It can promote the differentiation of adipocytes (in vitro). The overexpression in preadipocytes increases their proliferation, enhances adipogenesis, and reduces insulin-stimulated glucose uptake [54]. This result is also consistent with previous adipogenic differentiation experiments. Intriguingly, body-derived MSCs have a remarkable ability for proliferation and adipogenic differentiation in vitro. These differences may provide a basis for the selection of MSCs in later clinical treatment, and make accurate treatment possible.

Although dogs are important animal models and domestic companion pets, only a few studies have documented the comparison of the biological criteria and transcriptome profile of canine MSCs. Comparative experiments showed that AD-MSC, P-MSC, AM-MSC, and UC-MSCs have similar biological properties with BM-MSCs, such as fibrous adherent growth, expression of cell surface marker CD44, non-expression of CD34, and osteogenesis and adipogenic differentiation. Therefore, we suggested that in follow-up clinical trials, postpartum tissue-derived MSCs offers a better choice than BM-MSCs. The transcriptome sequencing results showed that the gene expression pattern of P-MSCs is significantly different from that of the other four types of MSCs, which may be connected to their biological functions used to nourish the fetus. Furthermore, body-derived MSCs and perinatal-derived MCSs had their own particular characteristics, to achieve accuracy in the treatment in which stem cell therapy is applied.

## 4. Materials and Methods

### 4.1. Animals

Dog adipose tissue, bone marrow, umbilical cord, placenta, and amniotic membrane were used in this experiment. All tissues were collected from the same adult Chinese rural dogs. All studies were approved by the Animal Ethics Committee of Foshan University, and were conducted in accordance with the ethical standards of the university. The project identification code is FSUeae2018006, and the approval date is 10 March, 2018.

### 4.2. Primary Culture of the Mesenchymal Stem Cell

For adipose mesenchymal stem cells (AD-MSCs), about 1 cm^3^; of adipose tissue was removed from the canine abdomen and washed with Phosphate Buffered Saline (PBS, Hyclon, Logan City, UT, USA) containing 5% Penicillin Streptomycin (Pen-Strep, Hyclon, America). The tissue was then minced and digested with 1 mg/mL of collagenase type I (Sigma, St.,Louis, MO, USA) at 37 °C for 1 h. After filtration through a 100-mesh cell strain, the filtrate was centrifuged for 5 min to collect AD-MSCs. The pellet was re-suspended into Dulbecco’s Modified Eagle Media (DMEM, Hyclon, Logan City, UT, USA) complete medium (DMEM basic medium with 10% fetal bovine serum, 1% Pen-Strep, and 1% L-glutamine) and transferred into cell culture dishes (Corning, New York, NY, USA).

For the bone marrow mesenchymal stem cells (BM-MSC), the canine bone marrow fluid was extracted aseptically into an anticoagulant tube and diluted with PBS. The dilution was loaded onto Ficoll solution in a centrifugation tube. After 20 min of centrifugation, the white cloudy layer formed by the nucleated cells was carefully sucked out, washed with DMEM, and centrifuged for 5 min. At last, the cell pellet was resuspended in complete medium, and transferred into cell culture dishes.

For the umbilical cord mesenchymal stem cells (UC-MSC), umbilical cord tissue was carefully isolated from postpartum discarded tissue of a female canine. Then, the UC-MSCs were obtained as AD-MSCs, with a prolonged digestion time of collagenase up to 6 h.

To obtain amniotic mesenchymal stem cells (AM-MSCs) and placenta mesenchymal stem cells (P-MSCs), amniotic membrane and placental tissue were carefully isolated from postpartum discarded tissue and minced into small pieces. Then, the tissues were incubated in 0.25% trypsin for 30 min in a 37 °C water bath before FBS was applied to stop the digestion. Again, the primary AM-MSCs and P-MSCs were isolated as described above.

All five MSCs were cultivated in DMEM complete medium containing 10% fetal bovine serum (FBS, BI, Kibbutz Beit-Haemek, Israel), 1% L-glutamine (Hyclon, Logan City, UT, USA), and Pen-Strep. The medium was changed after 48 h of primary culture, and then non-adherent cells were washed away with PBS. The medium was changed every 3 days, until the cells were overgrown for passage. The growth of the cells was observed daily.

### 4.3. Cell Growth Curve Production

P2, P5, and P8 of MSCs from 5 different sources were digested with trypsin and inoculated into 24-well plates at a concentration of 1 × 10^4^ cells/mL, and cultured at 37 °C in a 5% CO_2_ incubator. Three wells were randomly selected and counted after digestion “at the same time” each day for 8 days. The solution of the undigested wells was changed every 3 days. To create a growth curve, the number of days was plotted along on the abscissa and the number of cells on the ordinate.

### 4.4. Calculating Population Doubling Time

To compare the MSC proliferation rates, the following formula was used to calculate the cell population doubling time (PDT).

PDT = *t ×* [lg2/(lg*N_t_* − lg*N_0_*)]

where *t* is the time for cell culture (unit: h), *N*_t_ is the number of cells after the culture (*t*), and *N*_0_ is the number of cells initially inoculated [55].

### 4.5. Cellular Immunofluorescence Assay

The third-generation cells were inoculated on gelatin-coated 24-well culture plates. After adherence of the cells, the medium was discarded. Then the cells were washed twice with PBS containing 5% FBS, and fixed with 4% paraformaldehyde (Regal, Shanghai, China) at room temperature for 30 min. At last, immunofluorescence was performed to identify the expression of cell surface markers CD34 and CD44 (abcam, Cambridge, UK) [56].

### 4.6. Adipogenic and Osteogenic Differentiation

#### 4.6.1. Induction of Adipogenesis

P3 cells were seeded into 24-well plates at a density of 10^4^ cells/mL. The original medium was discarded when the cells reached 70% of confluence. DMEM adipogenic medium containing 10% fetal bovine serum, 1 μmol/L of dexamethasone, 10 μg/mL of insulin, and 200 μmol/mL of indomethacin (All from Cyagen, Suzhou, Jiangsu, China) was added into the cells. The fluid was replaced every 3 days. When the formation of lipid droplets in the cytoplasm was observed, the medium was carefully discarded. The cells were washed gently with PBS twice, fixed in 4% paraformaldehyde for 20 min, stained with oil red O (Solarbio, Beijing, China) for 30 min, and observed and photographed under a microscope. The time needed by each MSC to form a lipid droplet was recorded.

#### 4.6.2. Induction of Osteogenesis

P3 cells were seeded into 24-well plates at a density of 10^4^ cells/mL. The original culture medium was discarded the second day, and the DMEM osteoblast induction culture medium containing 10% fetal bovine serum, 0.1 μmol/L of dexamethasone, 10 mmol/L of sodium glycerophosphate, and 50 μmol/L of ascorbic acid (All from Cyagen, Suzhou, Jiangsu, China) was added for culture induction. The fluid was changed every 3 days. When bone calcification nodules were observed, they were identified with alizarin red (Solarbio, Beijing, China) staining. The time for each type of MSC to form calcium nodules was recorded.

### 4.7. Transcriptome Sequencing of Mesenchymal Stem Cells

The third generation of MSC cells, which were isolated from the umbilical cord, amniotic membrane, placenta, adipose tissue, and bone marrow-derived MSCs, were cultivated under the same conditions. When they reached 80% of confluence, Trizol lysis was used to lyse the cells.

The total cellular RNA was extracted by the Universal RNA Extraction kit (TaKaRa, Japan) and done according to the procedure of El-Ashram et al. [57]. After RNA quantization and purification evaluation [58], the following procedures were performed:

(1) The cDNA library for tanscriptome sequencing was prepared by the PCR method;
(2) The TruSeq PE Clustering Kit of v3-c Bot-HS (Illumia) was used to cluster the index coded samples on the c-bot clustering generation system;
(3) Purification of the raw data (raw readings) in FASTQ format was performed by an internal perl script, to obtain purified data of high quality for downstream analyses;
(4) Reference genome and gene model annotation files were downloaded directly from the genome website (https://www.ncbi.nlm.nih.gov/genome/85?genome_assembly_id=313739, accessed on: 9 August 2016). The index of the reference genome was constructed by Hisat2 v2.0.4, which was also used to compare the paired-end purified reads and the reference genome;
(5) For quantification of the expression level of genes, HTSeq v0.9.1 (Fabio Zanini, Stanford University, USA) was used to calculate the readings mapped to each gene. The FPKM of each gene was then calculated based on the length of the gene, and the readings localized to that gene were calculated too;
(6) The DEGSeq R software package (1.20.0) (Bioconductor, Stanford University, USA) was used for differential expression analysis, and the Benjamini and Hochberg method was used for the adjustment of the *p* value. A corrected *p* value of 0.005 and log2 (fold change) was set to 1, as a threshold for a significant difference expression.

### 4.8. Quantitative Reverse Transcriptase-PCR Analysis

By random selection, eight genes that were overexpressed or repressed from each group, and without differential expression, were chosen for verification by quantitative RT-PCR, which was performed as follows: the total RNA was extracted using a MiniBEST Universal RNA Extraction kit (TaKaRa, Japan, Code No:9767), and reverse-transcribed employing a PrimeScript RT reagent kit (TaKaRa, Japan, Code No:RR047A); an SYBR green-based RT-PCR was conducted by using TB Green Premix Ex Taq II (TaKaRa, Japan, Code No:RR820A), according to the instructions made by the manufacturer. The primers were synthesized by Invitrogen, Shanghai, China. The qRT-PCR primer sequences are described in Table 3. The results were expressed as fold changes [59]. A correlation analysis was performed between the RNA-Seq and RT-PCR fold change results of the same RNA samples before pooling, using Graphpad Software (San Diego, CA, United States). Additionally, experiments were conducted in triplicate, and data are displayed as mean ± SD [60].

## 5. Conclusions

We successfully isolated canine umbilical cord- adipose tissue-, amniotic membrane-, placenta-, and bone marrow-derived mesenchymal stem cells. All five cells showed fibrous adherent status, and expressed CD44 surface marker but not CD34. Five types of MSCs could differentiate into osteoblast and adipocyte. Among the five different sources of MSCs, AD-MSC had higher adipogenic differentiation efficiency than the other four types. Furthermore, AD-MSC had the best proliferative activity and the shortest population doubling time.

## Figures and Tables

**Table 1:** Test results of the expression of canine MSC surface marker genes.

Surface Marker	Gene Symbol	Gene Name	Gene ID	Expression in MSC
CD11a	ITGAL	integrin subunit alpha L	489905	-
CD11b	ITGAM	integrin subunit alpha M	489928	-
CD13	ANPEP	alanyl aminopeptidase, membrane	403913	+
CD14	CD14	CD14 molecule	607076	-
CD19	CD19	CD19 molecule	607898	-
CD33	CD33	myeloid cell surface antigen CD33	100686511	-
CD34	CD34	CD34 molecule	415130	-
CD44	CD44	CD44 molecule	403939	+
CD45	PTPRC	protein tyrosine phosphatase, receptor type C	490255	-
CD49a	ITGA1	integrin subunit alpha 1	489210	+
CD54	ICAM1	intercellular adhesion molecule 1	403975	+
CD73	NT5E	NT5E 5′-nucleotidase ecto	474984	+
CD86	CD86	CD86 molecule	403764	-
CD90	THY-1	Thy-1 cell surface antigen	489365	+
CD105	ENG	endoglin	609166	+
CD140a	PDGFRA	platelet derived growth factor receptor alpha	442860	+
CD140b	PDGFRB	platelet derived growth factor receptor beta	442985	+
CD146	MCAM	melanoma cell adhesion molecule	489368	-
CD271	NGFR	nerve growth factor receptor	491071	-
MHC I	DLA88	MHC class I DLA-88	474836	+
MHC II	DLA-DQA1	major histocompatibility complex, class II, DQ alpha 1	474861	-

Note: “+”indicates that gene has expression level(FPKM ≥ 1); “-”indicates that gene has no expression level (FPKM < 1).

**Table 2:** Differences in differentially expressed genes (FPKM) between body-derived MSCs and perinatal-derived MSCs.

Gene ID	Symbol	Gene Name	Gene Expression in Body-Derived MSCs (FPKM)		Gene Expression in Perinatal-Derived MSCs (FPKM)		
			BM-MSCs	AD-MSCs	UC-MSCs	AM-MSCs	P-MSCs
**down-regulated genes**							
476438	APOE	apolipoprotein E	19.48230703	173.6654375	1567.506632	7989.110095	546.5077788
474890	CDKN1A	cyclin dependent kinase inhibitor 1A	61.95423033	43.52563608	236.424318	1121.066431	218.6291872
487486	THBS1	thrombospondin 1	119.3758949	125.0178298	1524.233918	888.0058028	1051.135618
480221	RPL17	ribosomal protein L17	75.71858715	82.25616537	721.1519554	826.9961484	638.3271764
475792	ACTG2	actin, gamma 2, smooth muscle, enteric	6.399918524	0.414750909	351.1864758	766.518074	137.3140666
100856417	RASL11A	RAS like family 11 member A	0.876201127	1.40855383	680.0949342	629.3313239	673.6109411
611312	PHLDA1	pleckstrin homology like domain family A member 1	23.28421611	12.87378946	310.0875622	595.7103922	192.2748208
612596	DIO3	iodothyronine deiodinase 3	4.054952104	0	136.8582098	427.3538336	243.7423875
100271858	H19	H19, imprinted maternally expressed transcript (non-protein coding)	0.161668492	0.681456125	881.4958725	379.2382548	87.40665754
100855619	IGFBP3	insulin like growth factor binding protein 3	0.426966827	0.014979892	439.8998024	353.5566142	162.0637504
478256	FSIP1	fibrous sheath interacting protein 1	41.95676806	39.66236813	507.9665302	240.06563	353.193685
481519	TGFBI	transforming growth factor beta induced	22.9651713	35.26254056	200.9835386	178.8766204	205.2200716
**up-regulated genes**							
475230	TFPI2	tissue factor pathway inhibitor 2	1898.736999	266.7345781	4.765569024	32.96191818	44.31133656
490355	DPT	dermatopontin	309.4514262	236.9797415	27.20988994	21.23019758	57.24591462
478293	FBN1	fibrillin 1	289.0483951	397.5158581	87.97115218	47.3419576	54.44982126
609105	PRRX1	paired related homeobox 1	277.7767982	277.8458835	62.8372572	35.17884243	52.27789448
490458	S100A16	S100 calcium binding protein A16	134.3591612	141.5546125	1.853236286	28.06769912	11.59700716

**Table 3:** Primer sequences used for qRT-PCR.

Gene Symbol	Product Length	Temperature	Primer Sequence
APOE	163	59.49 °C	F: GGTGAAGATGGAGGAGCAGG R: CTTAGAGGTGGGGATGGTGG
COX1	198	58.93 °C	F: GGTCAGCCCGGTACTTTACT R: TGGAGGAAGGAGTCAGAAGC
HSP90B1	164	58.58 °C	F: GCAGTTTGGTGTCGGTTTCT R: TAATTGTTGTTCCCCGTCCG
HSP90A	194	58.95 °C	F: GTTCGGATGAGGAGGAGGAG R: TGCCAAGTGATCTTCCCAGT
IGFBP4	192	59.11 °C	F: CCGGAAAACAGGAGTGAAGC R: CCAGAGACAGAGCCAGGAC
PGS1	190	58.64 °C	F: CAGCCAGCAACCAATCACTA R: CCTTTCCCCAGCATTCACAC
PDGFRA	187	59.03 °C	F: ATCGAAGGCAGGCACATCTA R: TGTACCACCCCATCATTGCT
THBS1	160	59.09 °C	F: GCGCTCCTGTGATAGTCTCA R: GATCACACCATCACCACACG

## Supplementary Materials

Supplementary materials can be found at https://www.mdpi.com/1422-0067/20/6/1485/s1.

## Abbreviations

MSCs	Mesenchymal stem cells
AD-MSCs	Adipose Mesenchymal Stem Cells
AM-MSCs	Amniotic Mesenchymal Stem Cells
BM-MSCs	Bone Marrow Mesenchymal Stem Cells
P-MSCs	Placenta Mesenchymal Stem Cells
UC-MSCs	Umbilical Cord Mesenchymal Stem Cells
PDT	population doubling time
APOE	Apolipoprotein E
HSP90AA1	heat shock protein 90 alpha family class A member 1
IGFBP4	insulin like growth factor binding protein 4
THBS1	thrombospondin 1
PDGFRA	platelet derived growth factor receptor alpha
